# Massively parallel tag sequencing reveals the complexity of anaerobic marine protistan communities

**DOI:** 10.1186/1741-7007-7-72

**Published:** 2009-11-03

**Authors:** Thorsten Stoeck, Anke Behnke, Richard Christen, Linda Amaral-Zettler, Maria J Rodriguez-Mora, Andrei Chistoserdov, William Orsi, Virginia P Edgcomb

**Affiliations:** 1 Department of Ecology, University of Kaiserslautern, Kaiserslautern, Germany; 2 Université de Nice et CNRS UMR 6543, Laboratoire de Biologie Virtuelle, Centre de Biochimie, Parc Valose. F 06108 Nice, France; 3 Josephine Bay Paul Center, Marine Biological Laboratory, Woods Hole, MA, USA; 4 University of Louisiana at Lafayette, Lafayette, LA, USA; 5 Northeastern University, Boston, MA, USA; 6 Woods Hole Oceanographic Institution, Woods Hole, MA, USA

## Abstract

**Background:**

Recent advances in sequencing strategies make possible unprecedented depth and scale of sampling for molecular detection of microbial diversity. Two major paradigm-shifting discoveries include the detection of bacterial diversity that is one to two orders of magnitude greater than previous estimates, and the discovery of an exciting 'rare biosphere' of molecular signatures ('species') of poorly understood ecological significance. We applied a high-throughput parallel tag sequencing (454 sequencing) protocol adopted for eukaryotes to investigate protistan community complexity in two contrasting anoxic marine ecosystems (Framvaren Fjord, Norway; Cariaco deep-sea basin, Venezuela). Both sampling sites have previously been scrutinized for protistan diversity by traditional clone library construction and Sanger sequencing. By comparing these clone library data with 454 amplicon library data, we assess the efficiency of high-throughput tag sequencing strategies. We here present a novel, highly conservative bioinformatic analysis pipeline for the processing of large tag sequence data sets.

**Results:**

The analyses of ca. 250,000 sequence reads revealed that the number of detected Operational Taxonomic Units (OTUs) far exceeded previous richness estimates from the same sites based on clone libraries and Sanger sequencing. More than 90% of this diversity was represented by OTUs with less than 10 sequence tags. We detected a substantial number of taxonomic groups like Apusozoa, Chrysomerophytes, Centroheliozoa, Eustigmatophytes, hyphochytriomycetes, Ichthyosporea, Oikomonads, Phaeothamniophytes, and rhodophytes which remained undetected by previous clone library-based diversity surveys of the sampling sites. The most important innovations in our newly developed bioinformatics pipeline employ (i) BLASTN with query parameters adjusted for highly variable domains and a complete database of public ribosomal RNA (rRNA) gene sequences for taxonomic assignments of tags; (ii) a clustering of tags at k differences (Levenshtein distance) with a newly developed algorithm enabling very fast OTU clustering for large tag sequence data sets; and (iii) a novel parsing procedure to combine the data from individual analyses.

**Conclusion:**

Our data highlight the magnitude of the under-sampled 'protistan gap' in the eukaryotic tree of life. This study illustrates that our current understanding of the ecological complexity of protist communities, and of the global species richness and genome diversity of protists, is severely limited. Even though 454 pyrosequencing is not a panacea, it allows for more comprehensive insights into the diversity of protistan communities, and combined with appropriate statistical tools, enables improved ecological interpretations of the data and projections of global diversity.

## Background

Molecular surveys of protistan diversity research, traditionally based on amplification of small subunit (SSU) rRNA (SSU rRNA) gene fragments from environmental samples, clone library construction and Sanger sequencing have discovered protistan novelty at all levels of taxonomic hierarchy [1]. At the same time, such surveys indicated that we have described only a very small fraction of the species richness of protistan communities [2]. There are few SSU rRNA gene surveys of any community that are reasonably complete [3,4]; the majority appear to be no more than small samples from apparently endless lists of species present at any locale studied. (e.g. [1,2,5-9]). This is not only detrimental to the exploration of the true richness and complexity of protistan communities, but also hampers comparative analyses of protistan communities in an ecological and biogeographical context [10-12]. Massively parallel tag sequencing (454 sequencing, pyrosequencing) is a promising remedy and offers a means to more extensively sample molecular diversity in microbial communities [13]. For example Sogin et al. [14] analyzed up to 23,000 tags per sample of the V6 hypervariable region of the bacterial SSU rRNA genes from deepwater masses of the North Atlantic and hydrothermal vents in the NE Pacific. The study revealed that bacterial communities are one to two orders of magnitude more complex than previously reported, with thousands of low abundant populations accounting for most of the phylogenetic diversity detected in this study (the so called *rare biosphere*). This was confirmed by Huber et al. [15] who analyzed nearly 700,000 bacterial and ca. 200,000 archaeal V6 tag sequences obtained from two biogeochemically distinct hydrothermal vents. These data sets demonstrated that these distinct population structures reflect the different local biogeochemical regimes, corroborating previous indications that environmental factors and geographic separation lead to non-random distributions of microbes (see [16] for review, but see also [17]). Pyrosequencing has subsequently unveiled the richness and complexity of soil bacterial communities [18], human [19] and Macaque [20] gut microbiota. In the project described in this paper we applied the 454 sequencing technique to eukaryotes to analyze the complexity of microbial eukaryotic communities in two environmentally contrasting anoxic basins (Cariaco and Framvaren).

The Cariaco Basin is the world's largest truly marine anoxic body of water located on the northern continental shelf of Venezuela [21,22]. Primary production in Cariaco, microbial biomass, and midwater dark CO_2 _fixation vary strongly with factors such as seasonal riverine inputs, seasonal upwelling intensity, lateral intrusions of water from the Caribbean Sea, and trade-wind intensity [22-24]. The basin exhibits pronounced vertical chemical gradients controlled by physical transport of oxygen downwards and reduced compounds upwards countered by biological demands. Typically, oxygen concentrations decrease from saturation at the surface to 0 μM between 250 and 300 m. Deeper waters have remained anoxic and sulfidic down to the basin's bottom at ca. 1,400 m over timescales of centuries to millennia [25]. Significant enrichments in abundance of bacteria, bacterial activity and protists are routinely observed in the redoxcline and in the sulfidic waters underlying the redoxcline [23,26,27]. The Framvaren Fjord located in southwest Norway shares the feature of a defined oxic/anoxic interface with the Cariaco Basin. Yet, this fjord varies in many physico-chemical parameters (see Table 1) from the latter. For example, while the Cariaco Basin is truly marine with a redoxcline below the photic zone and relatively low sulfide concentrations below the redoxcline, the oxic-anoxic boundary layer of the fjord is located at shallow depth (ca. 18 m) with high sulfide concentrations below the redoxcline and steep biogeochemical gradients down to the bottom waters (180 m). Sulfide levels in bottom waters are 25 times greater than those in the Black Sea [28]. Initial studies of these two sites ([10,29,30]; Edgcomb et al. unpublished) based on clone-library construction and traditional Sanger sequencing indicate evidence for adaptation of protistan communities to differing environmental conditions along O_2_/H_2_S gradients. In spite of tremendous efforts in these previous studies, the sequencing depth was still significantly less than predicted total diversity and one might argue that additional sequencing would reveal homogeneous communities along these gradients. Massively parallel tag sequencing (in total, we analyzed 251,648 tag sequences obtained from the hypervariable V9 region of the SSU rRNA gene) offers the opportunity to evaluate if the structuring of microbial communities observed in these two contrasting basins still holds true at significantly increased sequencing efforts, whether richness predictions based on clone library analyses are supported and how well severely undersampled clone libraries reflect the "true" protistan diversity at a specific locale.

**Table 1:** Summary of recovery of pyrosequencing tags for Framvaren (FV) and Cariaco (CAR) samples, along with accompanying metadata.

	**Sample number-sample name**							
	**1-FV1**	**2-FV2**	**3-FV3**	**4-FV4**	**5-CAR1**	**6-CAR2**	**7-CAR3**	**8-CAR4**
N 454-reads total	38735	34171	24217	33962	35267	30277	28305	26714
Total eukaryotic tags > 100 bp	38280	32026	16256	32795	32876	22503	24266	23591
Unique eukaryotic tags	4280	5283	3765	5141	5983	5701	4325	4016
Total protistan tags (incl. Fungi)	23722	29402	12864	26543	30161	14453	7166	5969
Unique protistan tags (incl. Fungi)	3220	4825	3204	4439	5597	4616	2152	2070
Total and (unique) unassignable tags at 85%	1338	1153	1178	9189	2255	1724	1768	1042
	(276)	(468)	(427)	(758)	(620)	(580)	(556)	(365)
Total and (Unique) Archaeal tags	0 (0)	2 (2)	2(1)	2 (2)	0 (0)	4 (3)	6 (5)	2 (2)
Total and (Unique) Bacterial tags	0 (0)	0 (0)	1 (1)	0 (0)	0 (0)	0 (0)	0 (0)	0 (0)
Latitude/Longitude	58°09'N	58°09'N	58°11'N	58°09'N	10°30'N	10°30'N	10°40'N	10°30'N
	06°45'E	06°45'E	06°45'E	06°45'E	64°40'W	64°40'W	65°35'W	64°40'W
Temperature °C	10.7	8.4	8.1	5.8	17.9	17.7	17.6	17.6
Depth (m)	20	36	36	36	250	300	320	300
Salinity [80]	27	28	27.5	25.5	36.4	36.4	36.4	36.4
Nitrate (μmol/l)	---	---	---	---	5.22	nd	nd	0.02
Silicate (μmol/l)	---	---	---	100	31	39	41	43
Ammonium (μmol/l)	0.22	2.2	---	2.2	0.12	1.27	2.4	3.2
O_2 _(μmol/l)	nd	nd	nd	nd	nd	nd	nd	nd
H_2_S (μmol/l)	nd	668	362	600	nd	1.49	3.74	4.28
Bacteria (× 10^6 ^cells/ml)	4.6	0.78	0.43	0.61	0.487	0.149	0.18	0.244
Bact Production (H^3^-Leu, mg/m^2^/d)	670	160	----	----	347	353	1305	61
Chlorophyll a (μg/l)	1.22	nd	nd	nd	nd	nd	nd	nd
DNA conc (ng/μl)	160	170	170	120	5.58	4.55	9.12	10.34
Water volume sampled (/)	15	20	20	20	7	7	7	5
Sampling date	Sept-2005	Sept-2005	Sept-2005	May-2004	Jan-2005	Jan-2005	Jan-2005	May-2005

Nd = not detectable.

--- = not available

## Results

The number of high-quality eukaryotic reads we obtained from each sample ranged from 16,256 (FV3) to 38,280 (FV1). After dereplication (consolidating all sequences that are identical in primary structure into one OTU), the numbers of unique eukaryotic tags ranged from 3,765 (FV3) to 5,983 (CAR1). After exclusion of metazoan tags, we were left with numbers of unique tags ranging from 2,070 (CAR4) to 5,597 (CAR1), most of which could be assigned to protists and fungi (Table 1) for further analyses. The number of tags from non-eukaryotic domains was only marginal (0-0.02% of total tag reads, see Table 1) indicating the high domain-specificity of the primers used.

### Sampling saturation

**Figure 1:**
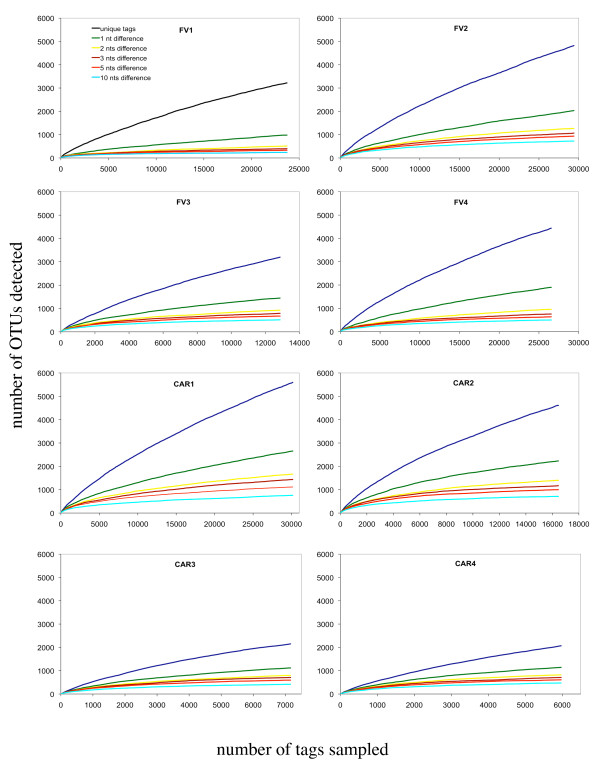
**Sampling saturation of V9 tag libraries**. Sampling saturation profiles of tag libraries generated for samples collected from anoxic waters of the Norwegian Framvaren Fjord (FV1-4) and the Caribbean Cariaco Basin (CAR1-4) at different levels of nucleotide differences for operational taxonomic units (OTUs). Only protistan and fungal tags were taken into account. Tags are clustered at k differences from k = 0 to 10 differences as described in pipeline 2 of the sequence data processing paragraph in the methods section. A difference can be an insertion or a mutation necessary to align the two sequences. At k differences, two tags having k or fewer differences are placed in the same cluster; if they have more than k differences, they are in two different clusters. Unique tags are tags clustered at 0 differences.

Despite substantial sequencing effort, the communities under study did not show saturation (Figure 1) in unique OTU richness. When clustering OTUs at one nucleotide difference, the number of OTUs detected decreased sharply, but still did not saturate. Only when clustering the tags at two, three, five and ten nucleotides difference (OTUs_xnt,_, where x is the number of nucleotide (nt) differences), did the sampling saturation profiles show a tendency of leveling off. The collapse of detected OTUs when comparing unique tags with OTUs based on two nucleotide differences (roughly 1.5% difference in primary structure), is remarkable: in the same sample (FV1) up to 6.3 times more unique OTUs were detected compared to OTUs_2 nt_. In contrast, the number of detected OTUs varied noticeably less when comparing OTUs over a clustering range of three to ten nucleotides, indicating that most of the tag variation was within two nucleotide differences between tags. Interestingly, regardless of the initial number of unique tags that varied greatly among the eight samples, all samples showed similar numbers of OTUs when tags were clustered at two, three, five and ten nucleotide difference.

### Rank abundance

**Figure 2:**
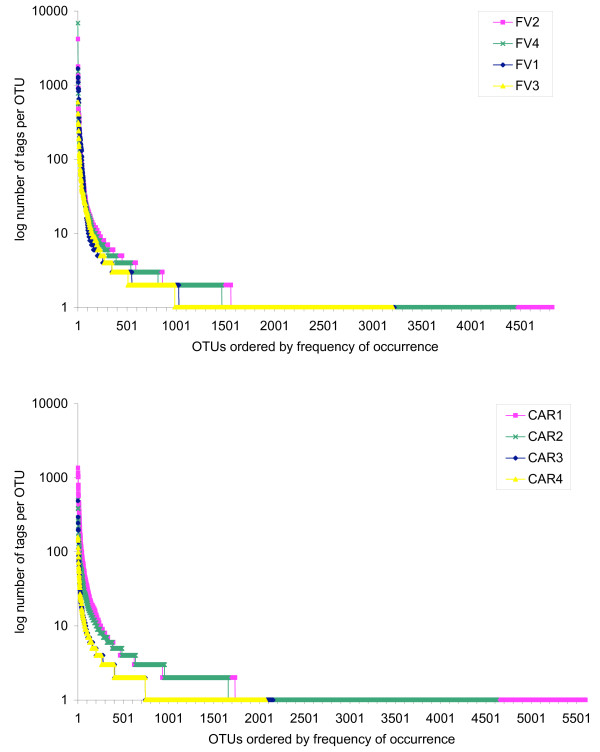
**Rank abundance distribution of unique protistan OTUs**. Protistan (including fungi) rank abundance distribution of unique operational taxonomic units (OTUs) obtained from four samples of the Caribbean Cariaco Basin (CAR1-4) and from four samples of the Norwegian Framvaren Fjord (FV1-4). For sampling sites information see Table 1. Curves were obtained when clustering tags at zero differences as described in pipeline 2 of the sequence data processing paragraph in the methods section. Subsequently tags were ordered according to decreasing rank (number of replicates present for each tag).

In all eight samples, the frequency distribution of protistan tags within unique protistan OTUs was very uneven (Figure 2): Only few populations were dominating the individual data sets, while the majority of OTUs contained less than ten sequences. The combined frequencies of these low-abundance unique phylotypes in the individual amplicon libraries accounted for 0.14%-0.03% of total protistan tags analyzed in each sample and thus, were considered as *rare*. Regardless of the sampling effort, this proportion of rare taxa remained similar for all samples (for example 96% rare populations in sample CAR4 and 95% in sample CAR1).

### Community comparisons

**Figure 3:**
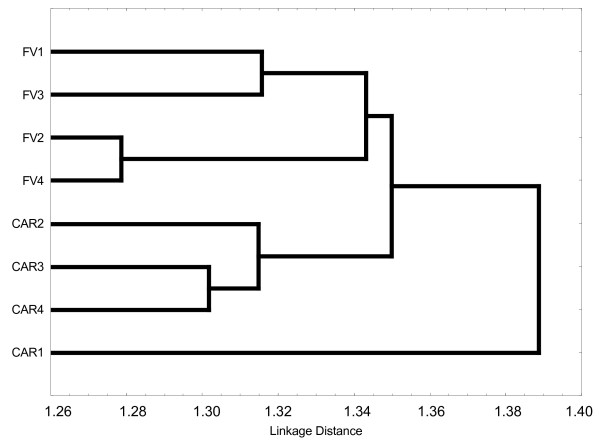
**Protistan community similarity in Cariaco and Framvaren samples**. Dendrogram resulting from calculated Jaccard index [27] based on incidence (J_incidence_) of unique operational taxonomic units (OTUs), as a measure of community similarity between the four Cariaco deep-sea samples (CAR1-4) and the four Framvaren Fjord samples (FV1-4) (for sampling sites information see Table 1). Jaccard similarity values were transformed into a distance matrix and subsequent cluster analysis was performed using the unweighted pair group mean average (UPGMA) algorithm. Details about calculation of this figure are described in the 'Community comparisons' paragraph of the methods section. Incidence data (presence/absence) of tags in each of the eight samples under study were obtained from a global tag-matrix as described in pipeline 3 of the sequence data processing paragraph in the methods section.

An UPGMA linkage distance analysis of unique OTUs based on J_incidence _(Figure 3) identified two distinct clusters one of which consisted of all FV samples, another of samples CAR4, CAR3 and CAR2, all from below the interface. The deep-sea sample from the Cariaco interface (CAR1) was the most distinct of all CAR samples regarding protistan community membership with higher affinity to the other CAR samples rather than to the FV samples. In the Framvaren Fjord, the two samples that were taken at different seasons from below the interface of the central basin were most similar to each other (FV2 and FV4), while the below-interface sample from the upper basin (FV3) - 3 km apart from the central basin station - was less similar to both FV2 and FV4. Neither samples CAR2 and CAR3, which were sampled from below the interface in the same season but at different locations, nor samples CAR2 and CAR4, which were sampled from below the interface at the same site but in different seasons clustered together. Instead, samples CAR3 and CAR4, were most similar in terms of community membership. These two samples were collected at two different seasons from below the interface at two different locations (Station B and Station A, respectively).

### Protistan community structures

**Figure 4:**
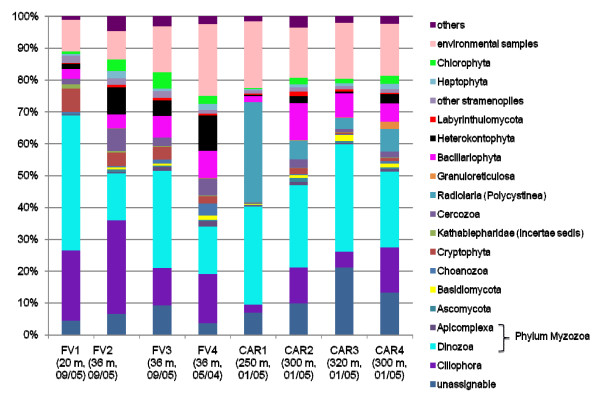
**Taxonomic distribution (phylum-based assignment) of protistan and fungal V9 tags**. Relative taxonomic distribution of unique protistan and fungal V9 tags generated from four anoxic water samples of the Caribbean Cariaco deep-sea basin (CAR1-4) and from four anoxic water samples of the Norwegian Framvaren Fjord (FV1-4). Phyla that were represented by a proportion ≥1% of all unique tags in at least one of the eight libraries used for 454 sequencing is shown. The category *others *denotes tags that could not be assigned to a taxonomic entity based on an 80% BLASTn similarity threshold and tags which fell into other phyla or taxon groups but were represented by <1% of the unique tags in all of the eight PCR amplicon libraries used for 454 sequencing. A higher resolution of lower-taxon rank-based assignments of dominant phyla is given in Figures 5-9. The data that served as a basis for the taxonomic bar chart are available as supplemental material (Table S3 in Additional file 5).

**Figure 5:**
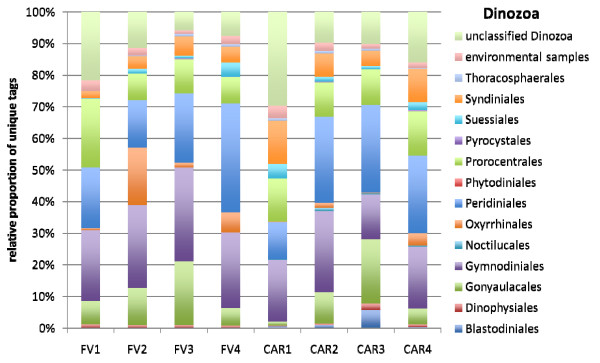
**Taxonomic distribution of V9 tags assigned to Dinozoa**. The data that served as a basis for the taxonomic bar chart are available as supplemental material (Table S4 in Additional file 5).

**Figure 6:**
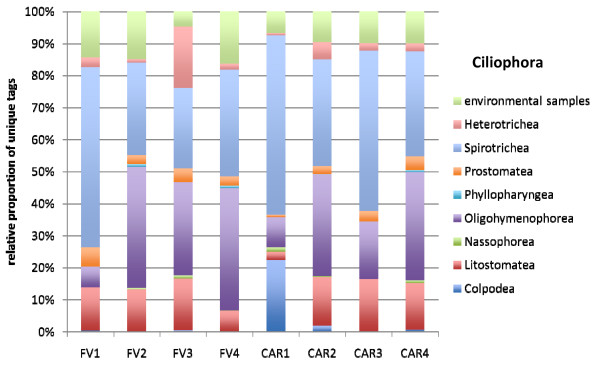
**Taxonomic distribution of V9 tags assigned to Ciliophora**. The data that served as a basis for the taxonomic bar chart are available as supplemental material (Table S5 in Additional file 5).

**Figure 7:**
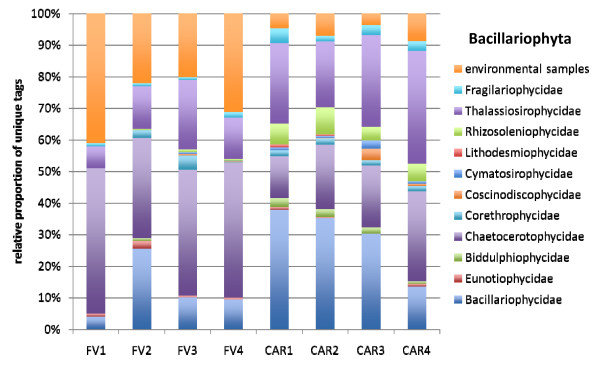
**Taxonomic distribution of V9 tags assigned to Bacillariophyta**. The data that served as a basis for the taxonomic bar chart are available as supplemental material (Table S6 in Additional file 5).

**Figure 8:**
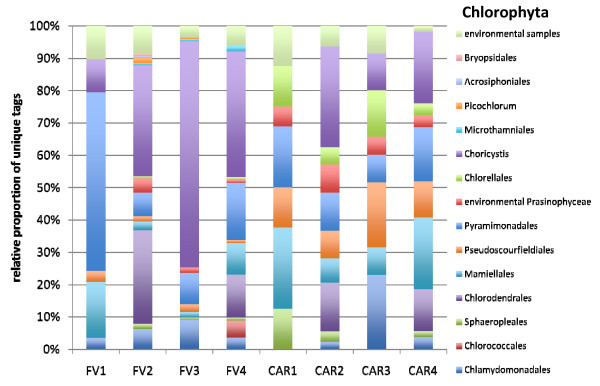
**Taxonomic distribution of V9 tags assigned to Chlorophyta**. The data that served as a basis for the taxonomic bar chart are available as supplemental material (Table S7 in Additional file 5).

**Figure 9:**
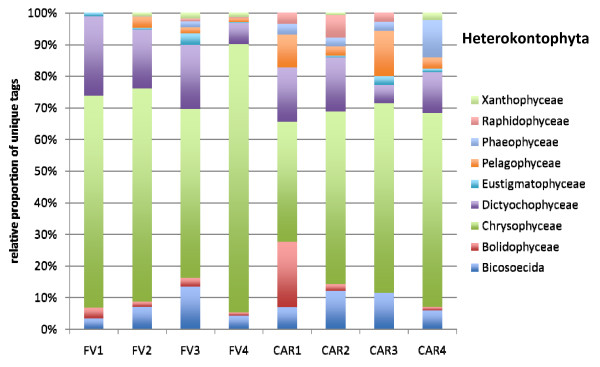
**Taxonomic distribution of V9 tags assigned to Heterokontophyta**. The data that served as a basis for the taxonomic bar chart are available as supplemental material (Table S8 in Additional file 5).

The vast majority of all unique tags could be confidently assigned to a defined taxonomic rank, at least at class-level (Figures 4, 5, 6, 7, 8 and 9). Between 3.5% (FV4) and 21% (CAR3) of unique tags could not be reliably assigned a taxonomic rank because sequence similarity to their best BLAST match was too low (<80%, see methods section). We attribute this to mainly two reasons. First, numerous sequences of described species that are deposited in GenBank lack the nucleotide positions that correspond to the V9 region of the SSU rRNA gene (ca. 1,620-1,790) in part or completely; second these unassignable tags correspond to as yet unsequenced taxonomic groups. Unfortunately it is currently not possible to discriminate between these two categories, rendering any interpretation of the proportion of unassignable tags speculative. We do not consider chimeras as a major contributor to unassignable tags because, as our protocol amplifies short DNA sequences with a negligible likelihood of chimera formation [31]. The proportion of unique tags that had only environmental sequences as the nearest match, without a sequence of a named species falling into the minimum 80% sequence-similarity boundary was large (up to 21% for sample FV4), reflecting the paucity of cultured representatives and the taxonomic annotation of environmental sequence data in public databases. In future studies, the implementation of specifically curated and annotated databases like KeyDNATools ([32] and ) will be beneficial for the taxonomic assignment of tags that have a good BLASTN match to environmental sequences but lack a species-match within a defined sequence similarity threshold. A tremendous number of higher taxonomic groups represented by tags that accounted for at least 1% of the overall number of protistan tags were discovered in each sample. For example, in sample FV3 we detected 17 such groups. When tag sequences that account for <1% of all protistan tags were taken into account (category 'others' in Figure 4), this number was even larger. Such groups included: Euglenozoa, Rhodophyta, Jakobida, Ichthyosporea, *Telonema*, *Cryothecomonas *and Apusozoa. In sum, all major eukaryotic lineages have been detected in each individual sample. However, the proportion of the different taxonomic groups in the individual samples varied considerably. Generally, all samples were dominated by alveolate OTUs, accounting for up to 64% of all unique protistan tags in an individual sample (FV1). In all CAR samples, Dinozoa contributed to the largest proportion of alveolate OTUs, followed by Ciliophora. The latter were noticeably less abundant in the CAR1 and CAR3 samples. In the Framvaren samples, Ciliophora comprised a decidedly larger proportion of the Alveolata, in FV4 and FV2 reaching or even exceeding the percentage of Dinozoa, respectively (Figure 4).

Interestingly, in nearly all dominant phyla occurring at both locales, the taxon composition in the non-sulfidic anoxic water depth was distinctly different from the sulfidic waters below the redoxcline (Figures 4a-f). For example, in the Cariaco Basin, unique tags assigned to Polycystinea accounted for 31% of all protistan tags in the interface (CAR1), while below the interface this number ranged between 3% (CAR3) and 7% (CAR4). However, generally, the genotype diversity in the sulfidic waters was decisively higher in a variety of taxon groups compared to the anoxic, non-sulfidic waters. Ciliophora tag proportion varied more than five-fold between CAR1 and CAR4, Cercozoa 18-fold between CAR1 and CAR2, Bacillariophyta seven-fold, Ascomycota nearly 10-fold, Heterokontophyta and Chlorophyta ca. seven-fold between these samples (Figure 4). This, even though to a lesser extent, was also true for the Framvaren Fjord. Just to mention a few examples, the proportion of Dinozoa-assigned tags decreased from 42% in the interface (FV1) to 14% below the interface (FV2), ascomycota increased nearly three-fold, Cercozoa increased ca. four-fold and Haptophyta, Chlorophyta and Heterokontophyta nearly five-fold (Figure 4). Figures 5, 6, 7, 8, 9 show the lower-rank taxonomic groups of dominant phyla displayed in Figure 4 that predominantly account for the differences in genotype richness between the non-sulfidic and the sulfidic water samples. While for example coscinodiscophycidae Bacillariophyta were missing from the non-sulfidic waters at both locales, they accounted for up to ca. 4% of all Bacillariophyta in the sulfidic waters (CAR3, Figure 7). This was also true for other taxonomic groups like Xanthophyceae and Phaeophyceae (Figure 9), Chlorodendrales (Figure 8); oligohymenophorean ciliates increased noticeably in the sulfidic waters at both locales (Figure 6), just to mention a few examples.

When comparing higher-taxonomic groups (phylum-level, Figure 4) found in Framvaren vs. Cariaco we noted that Radioloaria (all of which were exclusively Polycystinea), which are typically pelagic marine protists primarily found in the open ocean and very scarce or absent altogether in coastal waters [33], were the only higher taxon group that was detected in all Cariaco samples but in none of the Framvaren samples. Conversely, the proportion of Cercozoa in Framvaren samples was noticeably higher than in Cariaco. Differences between Framvaren samples and Cariaco samples become more pronounced when looking at lower taxon levels (Figures 5, 6, 7, 8, 9). For examples Blastodiniales and Noctilucales (Dinozoa, Figure 5) were exclusively found in all Cariaco samples. Rhizosoleniophycid Bacillariophyta (Figure 7) and Pseudoscourfieldiales (Chlorophyta, Figure 8) were noticeably higher in all Cariaco samples and Choricystis (Chlorophyta, Figure 8) genotypes were much more diverse in Framvaren.

## Discussion

The application of the 454 sequencing technique to the investigation of protistan communities in two anoxic marine basins revealed three significant findings. First, even a sampling effort that was one to two orders of magnitude larger than that achieved by environmental clone library construction and Sanger sequencing, was not successful in retrieving all unique SSU rRNA gene sequences present in a single sample (Figure 1). Up to 5,600 unique tags could be identified in a 7-L water sample from the Cariaco basin without reaching saturation (sample CAR1). However, this is unlikely to reflect the true species richness, because (i) not all SSU rRNA gene copies within a species are necessarily identical [34,35], (ii) some of the observed tag variability may be due to extreme variability of the V9 region in specific taxonomic groups, and [36] even when minimizing the effect of sequencing and PCR errors using a systematic trimming procedure (see Methods section and [14]) the accuracy of the 454 pyrosequencing strategy (GS-technology) is 99.75% - 99.5% for small subunit rRNA genes [37]. Indeed, in sample CAR1 the number of OTUs drops from 5,600 to ca. 2,600 when phylotypes are clustered based on one nucleotide difference (accounting for ca. 0.8% sequence similarity). Thus, about half of the unique protistan tags retrieved from this sample are potentially afflicted with an error and/or represent the same taxon. The detected number of unique tags would likely represent an overestimation of taxon richness. On the other hand, clustering OTUs at ten nucleotide differences (OTUs_10 nt_, reflecting ca. 8% sequence similarity) resulted most likely in an underestimation because different taxa may be lumped together into the same OTU. Consequently, it is reasonable to assume that the true taxon richness is reflected in the range between OTUs_1 nt _(ca. 1,700 in sample CAR1) and OTUs_5 nt _(ca. 1,200 in CAR1).

Interestingly, even the number of detected OTUs_10 nt _exceeded previous parametric and non-parametric richness estimates from the same sites, based on clone-library derived OTUs called at 99% or 98% sequence similarity, respectively [10,38,39]. Explanations for this may be several fold: (i) even though the sample sizes obtained from previous Cariaco and Framvaren clone libraries were relatively large, the sample size may still have been too small to obtain adequate resolution of the complex communities. If so, this makes previous clone library-based richness estimates severe underestimations; (ii) the statistical error of previous richness estimates may be too large, which cannot be assessed due to a lack of good confidence intervals; [36] abundance-based richness estimates may not reflect the true community richness or relative species abundance in a sample but rather the PCR-amplicon richness. The reasoning for the latter is that in contrast to bacteria, the copy number of SSU rRNA genes varies widely among protists [8,40,41]. Thus, the relative amplicon copy number after PCR does not necessarily reflect the relative abundance of a specific taxon in a sample, rendering abundance-based species richness estimates highly erroneous. It is likely that these factors and probably other factors that we cannot account for at present resulted in severe richness underestimations. We hypothesize that the protistan richness in marine anoxic waters by far exceeds previous estimates, and that anaerobic protistan communities are substantially more complex than previously reported. It will be interesting to further investigate how sequence divergence of a hypervariable SSU rRNA gene region translates into taxonomic entities. This will help interpreting the vast diversity of tags generated by massively parallel tag sequencing.

Most of the observed complexity was found in the low-abundance populations. Even when calling OTUs at five nucleotide differences, the proportion of rare OTUs (represented by less than 10 tags) ranges between 71% and 81% in FV samples and between 78% and 83% in CAR samples (data not shown), indicating that the high number of rare taxa is not an artifact based on high intra-species heterogeneity in the V9 region. This corroborates, to a somewhat lesser extent, the previous findings in the bacterial world [14,15,18]. The origin and meaning of this complexity is still unclear [42,43]. Actually, to date there is no evidence that this high frequency of low-abundant genotypes describes a true diversity. It could result from the amplification of detrital or extracellular DNA. On the other hand, it is reasonable to assume that a liter of water is only inhabited by a few individuals of a protist species that never meet in this volume and are therefore subjected to allopatric speciation. The result would be tremendous microheterogeneity that is reflected in these rare genotypes. One hypothesis suggests that these rare genotypes (if real) may represent a large genomic pool, which helps the protistan community to react to any biotic or abiotic changes [43]. In this *seed-bank *scenario, the species that are best adapted to prevailing environmental conditions would always be abundant in a community.

The second significant finding is the phylum-richness of protistan communities that is missed by the clone library/Sanger sequencing approach. Previous environmental protistan diversity surveys in the same sites of the Framvaren Fjord ([10] and Behnke et al. unpublished, accession numbers [DQ310187 to DQ310369 and EF526713 to EF527205]) did not retrieve any sequences assigned to Apusozoa, Chrysomerophytes, Centroheliozoa, Eustigmatophytes, hyphochytriomycetes, Ichthyosporea, Oikomonads, Phaeothamniophytes, and rhodophytes, all of which have been recovered with the massively parallel tag sequencing approach. Similarly, a vast array of higher taxon ranks detected in this tag-sequencing project could not be detected with an extensive clone library sampling in Cariaco ([26,30] Edgcomb et al. in preparation). Interestingly, the tags that could be assigned to taxonomic groups not detected via clone libraries all account for <1% of the unique protistan tags, explaining why they have been missed with the clone library approach [26,30]. Regarding taxonomic groups that were represented by large relative abundances of tags (e.g. alveolates and stramenopiles), the 454 data sets corroborate well with clone library-obtained data. Evidence of and tentative explanations for the dominance of these taxonomic groups in anoxic marine systems have already been intensively discussed elsewhere (e.g. [30,44,45]).

The broad taxonomic representation of 454 tags nicely demonstrates the efficiency of the primers used to target the hypervariable V9 region of eukaryote SSU rRNA genes. However, up to 50% of unique 454 tag sequences in our data sets were metazoa. This is a general problem also observed in SSU clone libraries (even though probably to a lesser extent) and not specific to 454 technology [46-48]. The consequence is that this large proportion of potential non-target tags has to be taken into account when designing protistan diversity studies using 454 technology. Either sequencing effort needs to be increased 1.5-fold to get the desired number of protistan tags, or group-specific 454 primers need to be applied subsequently to focus on selected protistan groups.

Our findings also reveal that higher sampling efforts can be obtained in a cost- and time-efficient way by the application of pyrosequencing, which therefore paints a substantially more comprehensive picture of protistan communities. The degree of undersampling inherent in most published clone library-based studies may be so high that it is possible that they cannot be compared in a meaningful manner to other equivalent surveys of diversity. Getting a comprehensive picture of a microbial community is critical to addressing fundamental questions in protistan ecology on the basis of molecular diversity surveys. Such questions include for example, determining the true richness and evenness of microbial communities, which is important in defining microbial ecosystem dynamics [15], and determining the biogeographic distribution of specific taxonomic groups, the stability of protistan communities over time, as well as local patchiness of protists. All of these community attributes are cornerstones for understanding microbial diversity, ecology, and evolution [16,49,50].

Some of these subjects frame the third important finding of this study. The eight sites sampled differed markedly in community composition. Based on community membership, it appears that protistan communities from the supersulfidic Framvaren Fjord with an interface located in the photic zone are distinct from the ones of a less sulfidic anoxic deep-sea site. Similarly, anaerobic protistan communities exposed to hydrogen sulfide are distinct from those that thrive in sulfide-free oxygen-depleted habitats. Even though we cannot unequivocally identify H_2_S as the single most important driving force shaping these protistan communities using this dataset, this observation is not unexpected: H_2_S-detoxification requires specific adaptation that is not necessarily present in all facultative or strictly anaerobic protists [51,52]. For example, Atkins et al. [53] found a significant difference in the hydrogen sulfide tolerance of different hydrothermal vent species they isolated, including the closely related sister taxa *Cafeteria *and *Caecitellus*. *Cafeteria *strains isolated by these authors could tolerate up to 30 mM sulfide under anoxic conditions over the 24 hr course of their experiment, *Rhynchomonas nasuta *could tolerate up to 5 mM sulfide, and *Caecitellus *could only tolerate up to 2 mM sulfide. Symbioses between protists and sulfide-oxidizing bacteria are another adaptive strategy observed in micro-oxic environments with high hydrogen sulfide concentrations. For example, the peritrich ciliate *Zoothamnium niveum *found in mangrove channels of the Caribbean Sea depends on its sulfur oxidizing ectobionts for detoxification of its immediate environment [54]. Scanning electron microscopy has revealed a visible diversity of ectobiotic prokaryotic associations with ciliates in the anoxic water column of Cariaco, and these associations are likely to be dependent on the distinct chemical nature of the basin's water column (see Additional file 1). The environmental selection pressure that acts on the phylogenetic composition of protistan communities can be of interest for the design of environment-specific phylo-chips (for example of application see Sunagawa et al. [55] that may help to monitor the global distribution of specific protistan communities.

The temporal and spatial resolution of our sampling strategy is insufficient to deduce temporal and spatial patterns in protistan communities under study. Yet, possible explanations for the observation that in the Cariaco deep-sea basin, samples collected from the same depth at two different points in time are distinctively less similar to each other (samples CAR2 and CAR4 in Figure 3, UPGMA), compared to the shallow Framvaren Fjord (samples FV2 and FV4) are obvious: Surface waters of the Cariaco Basin are subject to strong seasonal upwelling, driving as much as 13-fold excursions in net primary production (NPP) between upwelling and non-upwelling seasons [22]. This causes significant seasonal variations in vertical carbon fluxes, which seems to be not only very important for the dynamics of viral [27] and bacterial communities [56] in such systems, but also for protistan communities, even though the exact mechanisms for how vertical carbon flux variations may act on protistan communities are largely unknown. One possibility could be that due to selective interactions of protist with specific bacteria [57-59], changes in vertical carbon flux that have a direct influence on bacteria can act indirectly on protistan communities.

**Figure 10:**
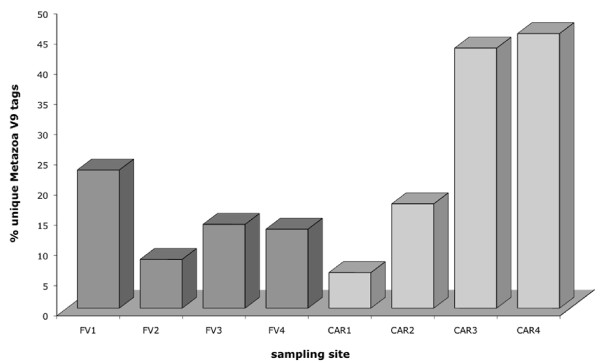
**Proportion of unique eukaryote tags assigned to metazoa in the individual amplicon libraries**. For library designation see legend of Figure 1.

At first glance it seems disturbing that metazoa accounted for up to ca. 50% of all eukaryote tags (Figure 10). Because most metazoans are very sensitive to anoxia and hydrogen sulfide, this raises the question about the nature of these tags, whether they represent organisms that could plausibly live in the geochemical environments under study or rather represent contamination. Such high proportions of unique metazoan tags are indeed not unexpected after careful consideration: body parts, eggs or planktonic larvae of an individual taxon that may have been present in 5 to 10 liter water samples used for DNA extraction would contribute tremendous amounts of genomic DNA compared to the few individuals of a protistan taxon. Therefore, the SSU rRNA gene copies of this individual metazoan taxon would outnumber any protistan SSU rRNA gene copy numbers by far, resulting in high proportions of metazoan tags. For example, one individual copepod contributes almost 9,000 nearly identical amplicons to the FV1 amplicon library (Additional file 2). In order to account for intrinsic error rates of the pyrosequencing technique (see above) and for intraspecies SSU rDNA polymorphisms as described above for protistan data, we also clustered all metazoan tags at one to five nucleotides differences in a separate analysis. Indeed, it turned out that the proportion of unique metazoan tags decreased decisively (Additional file 3), accounting for only 3.9% to 11.4% (Additional file 4) of total eukaryote tags when clustered at five nt differences (ca. 2% sequence divergence). Data serving as the basis for the relative distribution of taxonomic groups presented in Figures 4-9 can be found in Additional file 5.

Only a few taxa accounted for most of these metazoan tags, which belonged predominantly to copepods, cnidaria, ctenophores, molluscs and polychaetes (Additional file 2). Copepods can survive anoxia and high hydrogen sulfide concentrations for long periods of time [60]. Also several molluscs [61], cnidarians, ctenophores [62] and polychaetes are tolerant of anoxia [63]. Even Bryozoa that were detected in three of the samples (Additional file 2) are capable of thriving under anoxic conditions [64]. Thus, the detection of metazoan sequences in anoxic environments retrieved by domain (Eukarya)-specific PCR primers is not surprising. Yet, with the exception of copepods, which we can observe frequently at least in the oxic-anoxic interfaces of our sampling sites we did not confirm the presence of these metazoan taxa in the water samples under study by visual inspection. This is mainly due to the fact that we only screened 20-μl aliquots microscopically (for protistan target taxa). Because of this, small forms (life stages) of larger metazoans or small metazoans like bryozoa represented in our amplicon libraries may have been easily overlooked. It is reasonable to assume that the metazoan amplicons may represent a mixture of allochtonous material (see the detection of an hymenoptera phylotype in FV4 that is represented by nearly 5,000 amplicons) and autochtonous organisms. However, taking into account the low proportion of unique metazoan taxa when clustered at 5 nt differences and the high likelihood of the indigenous nature of most of the metazoans represented by the non-protistan tags it is reasonable to consider contamination in general as an insignificant issue.

This study shows that when 454 pyrosequencing of the V9 region is paired with rigorous downstream data processing, this method is more time- and cost-efficient, and produces a much more comprehensive picture of the protist community than Sanger sequencing of clone libraries, allowing for better estimates of community complexity. While direct comparison of the Framvaren and Cariaco communities is complicated by multiple physico-chemical differences between these two sampling locations, it is possible to distinguish protistan communities on the basis of community composition in the supersulfidic Framvaren Fjord with an interface located in the photic zone from those in the deep-sea anoxic and less sulfidic environment. Furthermore, protistan populations in the sulfide-free oxic/anoxic interface in both Framvaren and Cariaco are distinct from those that are exposed to hydrogen sulfide. However, the specific environmental factors structuring protistan communities remain unknown.

## Conclusion

This study combined high-throughput parallel tag sequencing with a highly conservative bioinformatic analysis pipeline to investigate protist community complexity in two contrasting anoxic marine ecosystems (Framvaren Fjord, Norway and Cariaco Basin, Venezuela). Our data suggest that both ecosystems under study are highly variable regarding the dynamics of protistan communities on a spatial and temporal scale. However, high-resolution sampling will be necessary to reliably assess the true extent of this variability. Furthermore, this study illustrates that our current understanding of the ecological complexity of protist communities, and of the global species richness and genome diversity of protists, is severely limited. A deep sequencing of ca. 250,000 V9 SSU rRNA gene tags in total allowed us to recover eukaryotic taxonomic groups that were not detected by previous clone library-based diversity surveys of both sampling sites. Furthermore, the number of detected OTUs far exceeded previous richness estimates from the same sites based on clone libraries and Sanger sequencing, even when tag sequences were clustered at up to ten nucleotide differences (reflecting ca. 8% sequence similarity). Our data highlight the magnitude of the under-sampled *protistan gap *in the eukaryotic tree of life, and support the notion that not only in bacteria but also in protists over 90% of the observed diversity was represented by rare OTUs that had less than 10 sequence tags assigned to them. Even though 454 pyrosequencing is not a panacea, it allows for more comprehensive insights into the diversity of protistan communities, and combined with appropriate statistical tools, enables improved ecological interpretations of the data and projections of global diversity.

## Methods

### Sampling sites and collection procedure

Samples were collected from two locales, the Cariaco Basin, located on the northern continental shelf of Venezuela, and the Framvaren Fjord located in Southwest Norway. Sampling protocols for both sites were as described elsewhere, as well as the protocols for measurement of physico-chemical and biological parameters [10,26]. Depths of samples, volumes of water collected, and physicochemical characteristics at the time of sampling are presented in Table 1. The sampling design accounted for three features: (i) temporal effects (same site sampled at different seasons), (ii) local patchiness (same depth sampled at two distant sites at each locale), [36] environmental factors (vertical water column gradient at each site and distinct locale-characteristics). Cariaco samples were collected at the Cariaco Time Series Station A (10.30°N, 64.40°W) and at Station C (10.40°N, 65.35°W). These two stations are located in the eastern and western sub-basins of the Cariaco system, respectively. Cariaco samples were collected from Station A at the depth corresponding to the oxic/anoxic interface (250 m, oxygen and sulfide not detectable) in January 2005 (CAR1) and from below the interface (300 m) in January 2005 (CAR2) and May 2005 (CAR4). Sample CAR3 was collected at Station C from below the interface (320 m) ca 100 km distant from Station A, in January 2005. Framvaren Fjord samples were collected in the central basin (58.09°N, 06.45°E) from the oxic/anoxic interface at 20 m sampled in September of 2005 (FV1, oxygen and sulfide not detectable), and from below the interface (36 m) in September 2005 (FV2) and May of 2004 (FV4). The sample FV3 was taken in the upper basin (58.11°N, 06.45°E, ca. 3 km distant from the sampling location in the central basin) from below the interface at 36 m in September 2005.

### DNA isolation, PCR amplification, and 454 pyrosequencing

DNA was isolated from environmental samples and quality-checked as described previously [26]. In short, samples were taken with Niskin bottles and drawn onto 0.45 μm Durapore membranes (Millipore, Billerica MA, USA) under anoxic conditions with no prefiltration step. Samples were frozen immediately in liquid nitrogen until further processing in the laboratory. The nucleic acid extraction protocol employed a high-salt extraction buffer (100 mM Tris HCl (pH 8), 100 mM sodium phosphate buffer (pH 8), 1.5 M NaCl, 100 mM EDTA (pH 8.)) with 1% cetyl trimethylammonium bromide. Approximately 3 ml of this buffer was added to one filter and the total genomic DNA was extracted using chloroform-phenol extraction and isopropanol precipitation. In order to minimize bias caused by sampling the extracts from three filters per sample site were combined prior to polymerase chain reaction-amplification. Our strategy targeted the V9 hypervariable region of the SSU rRNA genes [65]. This region was chosen because it is (i) among the most variable of eukaryotic SSU rRNA hypervariable regions [66], represents a good marker for the taxonomic complexity of protistan communities, (ii) allowed for the use of conserved PCR-primers that target most described major eukaryote lineages, [36] has only marginal length variability among different taxonomic groups (127-150 bp) and (iv) could be fully sequenced using the Roche GS FLX system (up to 250 bp-reads) developed by 454 Life Sciences ([65], Stoeck T., Richards T, and Bass D., unpublished). PCR amplification and pyrosequencing followed the protocol of Amaral-Zettler et al. [65]. The PCR primers we used flanked the V9 region of eukaryote SSU rRNA genes. These primers were 1,380F (forward 1), 1,389F (forward 2), and 1,510R (reverse). Separate 1380F/1510R and 1389F/1510R reactions were run for each sample to recover the broadest eukaryotic diversity possible. The 454 Life Science's A or B sequencing adapters were fused to the 5' end of the primers. For each individual environmental DNA extract we ran three independent 30-μl PCR reactions with reaction mix consisting of 5 U of Pfu Turbo polymerase (Stratagene, La Jolla, CA, USA), 1× Pfu reaction buffer, 200 μm dNTPs (Pierce Nucelic Acid Technologies, Milwaukee, WI, USA), a 0.2 μM concentration of each primer in a volume of 100 μl, and 3-10 ng genomic DNA as template. The PCR protocol employed an initial denaturation at 94°C for 3 min; 30 cycles of 94°C 30 s, 57°C for 45 s, and 72°C for 1 min; and a final 2 min extension at 72°C. PCR products from the same DNA sample were pooled and cleaned by using the MinElute PCR purification kit (Qiagen, Valencia, CA, USA). The quality of the products was assessed on a Bioanalyzer 2100 (Agilent, Palo Alto, CA, USA) using a DNA1000 LabChip (Agilent). Only sharp, distinct amplification products with a total yield of >200 ng were used for 454 sequencing. The fragments in the amplicon libraries were bound to beads under conditions that favor one fragment per bead. The emulsion PCR (emPCR, [67]) was performed by emulsifying the beads in a PCR mixture in oil, with PCR amplification occurring in each droplet, generating >10 million copies of a unique DNA template. After breaking the emulsion, the DNA strands were denatured, and beads carrying single-stranded DNA clones were deposited into wells on a PicoTiter-Plate (454 Life Sciences) for pyrosequencing on a Genome Sequencer FLX system (Roche, Basel, Switzerland) at the Marine Biological Laboratory (Woods Hole, MA, USA). In total, we recovered 251,648 sequence reads for the eight samples that were subjected to quality control. Removal of low quality sequences [14] left us with 222,593 high-quality reads for further consideration. Tag sequences have been deposited in the National Center for Biotechnology Information (NCBI) Short Read Archive (SRA) under the accession number SRP001212.

### Sequence data processing

We developed three automated analysis pipelines to analyze quality-checked 454 reads: The first pipeline for taxonomic assignment of V9 tags, the second pipeline for clustering V9 tags at different sequence similarity levels and dereplication, and the third pipeline to construct a global tag-matrix for sample comparison.

#### 1.) Taxonomic assignment of V9 tags

The first pipeline was aimed at assigning taxonomy to our 454 tags and included four steps. First, 454 reads were preprocessed to remove reads with more than 1 ambiguity (N) and short sequences (having fewer than 100 nucleotides after the proximal primer), as well as all sequences having mismatches with the PCR primers. Second, each remaining sequence was compared through similarity searches, using the program BLASTN (version 2.2.21, [68]), against a reference database including every SSU rRNA sequence longer than 800 nt (561,000 sequences) extracted from 1,300,000 SSU rRNA genes present in the EMBL/GenBank database, with three longest sequences selected to represent each family (as described in their respective EMBL entries). This served to remove tags that matched with at least 70% similarity to sequences from Archaea, Bacteria or Metazoa. Third, the remaining sequences were blasted against all publicly available SSU rRNA gene sequences of protists, fungi and viridiplantae (170,000 sequences), requesting up to 150 best hits, using the BLAST parameters: -m 7 -r 3 -q -2 -G 6 -E 6. Parameter -m 7 allowed for an XML output, which was easier to analyze. The other parameters were selected after running 1,500 test BLAST runs using tags extracted from longer, well known sequences in order to finely tune the blast search to the characteristics of the domain analyzed. Fourth, the blast output was parsed to extract *Best *and *Highest *hits at a series of thresholds for sequence similarity. Sequence similarity was calculated as the sum of identities for non-overlapping (if any) HSP (High Scoring Pairs, see the BLAST documentation) divided by the length of the query sequence; this is a much more efficient method than simply taking the first HSP into account as is usually done. *Best hit *was the most similar target sequence that had a good taxonomy associated with the sequence (i.e. the Organism Classification (OC) field in the EMBL entries). The *Highest Hit *was the sequence with the highest similarity overall. Also, every sequence above the designated threshold was used to build a list of taxa (i.e. the contents of the OC field in the EMBL entries), which allowed for verification of whether the taxonomic assignment of the best hit was in global agreement with the next most similar sequences. Results at thresholds of 70, 75, 80, 85, 90, 95, 98 and 99% similarity were stored as tabulated files for further analyses. A manual examination of the relationships between threshold and qualities of taxonomic assignment led us to choose the 80% similarity threshold for assigning a given tag to a taxon (see the results). The reasoning for this similarity threshold is based on GenBank sequence data analyses. Therefore, we extracted the V9 regions from a random selection of 100 full-length eukaryote SSU rRNA gene sequences with a described taxonomy. A BLASTN analysis of the V9 fragments against the GenBank nr database revealed that the short V9 fragments could reliably be assigned to order-level when the closest BLAST hit (the original respective full-length sequence excluded) was at least 80% (see also [33,34]). Taxonomy of protists is according to Adl et al. [69] and for fungi according to Hibbett et al. [70]. We note that because Synurophyceae and Chrysophyceae are hardly distinguishable even when full-length 18S rRNA gene sequences are available, we united tags that were putatively assigned to Synurophyceae with Chrysophyceae to Heterokontophyta.

#### 2.) Similarity clustering of 454 tags and dereplication

The second pipeline was dedicated to the clustering of tags at a given level of similarity. This is usually done by first using a multiple sequence alignment (MSA) program (usually MAFFT [71] or MUSCLE [72] to align the tags, followed by the calculation of a distance matrix (using QuickDist [14] for example) and finally statistical analyses. Our experience with the V9 domain indicated that none of the MSA programs was able to output alignments of high enough quality. We therefore implemented a completely new approach (Shahbazkia & Christen, in preparation). Our key hypothesis was that the greater frequency at which a given sequence occurs, the more likely it represents a *real *sequence. Conversely, there is a probability that a sequence found only once is the result of a PCR or sequencing error, or due to the presence of variations in some operons within a single genome [73]. First a python program allowed for a strict dereplication, i.e. clustering strictly identical sequences. This led to a 5 to 10 fold reduction in the number of sequences. Strict dereplication allowed for the second step, but also allowed for the construction of rank abundance curves. The resulting file (of strictly dereplicated tags) was sorted by decreasing abundances of tags in each cluster. Then, instead of computing a percentage of similarity between sequences (which is difficult because we don't know how to implement a good substitution matrix for hypervariable regions of rRNA sequences) we implemented a Levenshtein distance calculation for clustering sequences. Levenshtein distance [74] is a measure of the similarity between two strings, which we will refer to as the source string (s) and the target string (t). The distance is the number of deletions, insertions, or substitutions required to transform s into t. Taking successively each dereplicated tag, the following tags were clustered with this representative if they had a Levenshtein of k or less (k ranging from 1 to 10). A number of checks were performed to analyze such clusters. A comparison of these clusters to the taxonomic assignments performed by the first pipeline showed an almost perfect agreement when taxonomic assignments had been possible by BLAST (k = 1,2,3). Above these k values many non-assigned tags could be assigned to clusters containing assigned tags.

Comparisons of operational taxonomic units (OTUs) based on V9 domains and (almost) complete SSU rRNA sequences are almost impossible on large data sets of sequences because none of the multiple sequence alignment software is able to properly align SSU rRNA sequences within their divergent domains, and this problem is exacerbated for short divergent tag sequences (Guillou & Christen unpublished). For this reason, published 454 studies have relied heavily on BLAST alignment to public sequences to cluster tags. We used a completely new algorithm (Shahbazkia & Christen, unpublished) that directly clusters tag sequences having less than k differences (k = 0, 1....10) and does not rely on a multiple sequence alignment. We validated this approach in a separate analysis (Guillou and Christen unpublished) by demonstrating that our tag clustering method based on word counting instead of percent sequence similarity identified correctly the almost full-length sequences of a separate large, well-curated SSU rRNA alignment from which tags were extracted, and that using the clustering approach here, the same cluster ID was attributed to sequences that were phylogenetically close to the original tag sequence (Guillou and Christen, unpublished).

#### 3.) Tag matrix for sample comparison

A final pipeline was designed for the global statistical analysis of all eight samples. The entire data set consisting of all eight samples was this time considered and globally treated as described above in pipelines 2 and 3. This led to the construction of an *abundance matrix *at various clustering values as explained above where each column was a given sample and each line a cluster, values being either the number of occurrences of the tag in the sample, or simply 1 or 0 to indicate presence or absence of sequences belonging to that cluster. However, we here refrained from further analysis of the abundance-matrix, because due to different genome sizes and rRNA gene copy numbers among protists [75] and PCR primer selectivity [30] the abundance of PCR-amplicons from a sample does not necessarily reflect the relative abundance of the respective organisms in this sample.

The script for data analyses (Linux, Windows and Macintosh operating systems) is provided online .

### Community comparisons

We calculated the Jaccard index, based on incidence (J_incidence_) of unique OTUs as obtained from the third data processing pipeline described above, as a measure of community similarity between the eight samples under study using the program package SPADE [76]. Analyses were performed as recommended by the authors. Similarity values were transformed into a distance matrix and used for an Unweighted Pair Group Method with Arithmetic Mean analysis (UPGMA) of the eight unique libraries [77].

Data from the authors cited as unpublished are available from the authors upon request.

## Abbreviations

OUT: operational taxonomic unit; PCR: polymerase chain reaction; MSA: multiple sequence alignment; DNA: deoxyribonucleic acid; RNA: ribonucleic acid; UPGMA: Unweighted Pair Group Method with Arithmetic Mean; NPP: net primary production; NE: northeast; CAR: Cariaco Basin; FV: Framvaren Fjord; BLAST: Basic Local Alignment Search Tool; SSU rRNA: small subunit ribosomal RNA.

## Authors' contributions

TS, VE, RC and AB conceived and designed the experiments. TS, VE, RC, AB and MJR-M performed the experiments. TS, VE, RC, AB, WO and LAZ analyzed the data. RC contributed analysis tools. VE, TS, AB and LAZ wrote the paper.

## Supplementary Material

Additional file 1 **Scanning electron micrograph of an unidentified ciliate isolated from anoxic, sulfidic waters of the Cariaco Basin**. Figure S1. The ciliate in the picture, isolated from anoxic waters of the Cariaco basin, is covered with bacterial ectosymbionts. Protists with bacterial ectosymbionts are frequently recovered from sulfidic waters of both, the Cariaco Basin as well as the Framvaren Fjord. It is not unlikely that these as yet unidentified bacteria may play a role as an adaptive mechanisms for some protists to thrive in anoxic sulfidic environments. This picture is courtesy of Orsi W., Edgcomb V., Hohemann T. and Epstein S.S. as part of a study on bacterial ectosymbionts on protists from the Cariaco Basin (Orsi et al., in preparation for publication). Click here for file

Additional file 2 **Taxonomy and proportion of abundant metazoan operational taxonomic units**. Table S1. Taxonomy and proportion of abundant metazoan operational taxonomic units (OTUs) accounting for at least 1% of all metazoan OTUs of a specific amplicon library from four anoxic water samples from the Caribbean Cariaco deep-sea basin (CAR1-4) and four anoxic water samples of the Norwegian Framvaren Fjord (FV1-4). OTUs were established based on identical best GenBank hit. For each OTU the best GenBank match is given (accession no., organism description, and taxonomy), as well as the number of total and unique tags. Unique tags are tags clustered at 0 differences. Click here for file

Additional file 3 **Numbers of unique metazoan operational taxonomic units**. Figure S2. Number of unique metazoan operational taxonomic units (OTUs) obtained from four samples of the Caribbean Cariaco Basin (CAR1-4, Figure S2-A) and four samples of the Norwegian Framvaren Fjord (FV1-4, Figure S2-B) at different levels of nucleotide differences. Tags were clustered at nt differences zero to five differences as described in pipeline 2 of the sequence data processing paragraph in the methods section. A difference can be an insertion or a mutation necessary to align the two sequences. At k differences, two tags having k or fewer differences are placed in the same cluster; if they have more than k differences, they are in two different clusters. Click here for file

Additional file 4 **Relative contribution of metazoan operational taxonomic units to total eukaryote operational taxonomic units**. Table S2. Relative contribution of metazoan operational taxonomic units (OTUs) to total eukaryote OTUs when clustered at 5 nt differences (OTUs_5 nt_) in the Franvaren Fjord (FV1-FV4) and the Cariaco Basin (CAR1-CAR4) amplicon libraries. Click here for file

Additional file 5 **Relative taxonomic distribution of unique protistan and fungal V9 tags**. Table S3. Accompanying data to Figure 4. Relative taxonomic distribution of unique protistan and fungal V9 tags generated from four anoxic water samples of the Caribbean Cariaco deep-sea basin (CAR1-4) and from four anoxic water samples of the Norwegian Framvaren Fjord (FV1-4). Phylum-based assignment; phyla that were represented by a proportion ≥1% of all unique tags in at least one of the eight libraries used for 454 sequencing is shown. The category "others" denotes tags that could not be assigned to a taxonomic entity based on an 80% BLASTn similarity threshold and tags which fell into other phyla or taxon groups but were represented by <1% of the unique tags in all of the eight PCR amplicon libraries used for 454 sequencing. Table S4. Accompanying data to Figure 5. Relative taxonomic distribution of unique protistan and fungal V9 tags generated from four anoxic water samples of the Caribbean Cariaco deep-sea basin (CAR1-4) and from four anoxic water samples of the Norwegian Framvaren Fjord (FV1-4) within the Dinozoa. Table S5. Accompanying data to Figure 6. Relative taxonomic distribution of unique protistan and fungal V9 tags generated from four anoxic water samples of the Caribbean Cariaco deep-sea basin (CAR1-4) and from four anoxic water samples of the Norwegian Framvaren Fjord (FV1-4) within the Ciliophora. Table S6. Accompanying data to Figure 7. Relative taxonomic distribution of unique protistan and fungal V9 tags generated from four anoxic water samples of the Caribbean Cariaco deep-sea basin (CAR1-4) and from four anoxic water samples of the Norwegian Framvaren Fjord (FV1-4) within the Bacillariophyta. Table S7. Accompanying data to Figure 8. Relative taxonomic distribution of unique protistan and fungal V9 tags generated from four anoxic water samples of the Caribbean Cariaco deep-sea basin (CAR1-4) and from four anoxic water samples of the Norwegian Framvaren Fjord (FV1-4) within the Chlorophyta. Table S8. Accompanying data to Figure 9. Relative taxonomic distribution of unique protistan and fungal V9 tags generated from four anoxic water samples of the Caribbean Cariaco deep-sea basin (CAR1-4) and from four anoxic water samples of the Norwegian Framvaren Fjord (FV1-4) within the Heterokontophyta. Click here for file
