# Supplementary figures and images for: Massively parallel tag sequencing reveals the complexity of anaerobic marine protistan communities
Source: BMC Biol. 2009 Nov 3;7:72. doi: 10.1186/1741-7007-7-72 (PMC2777867; doi:10.1186/1741-7007-7-72)

Supplemental Figure S1

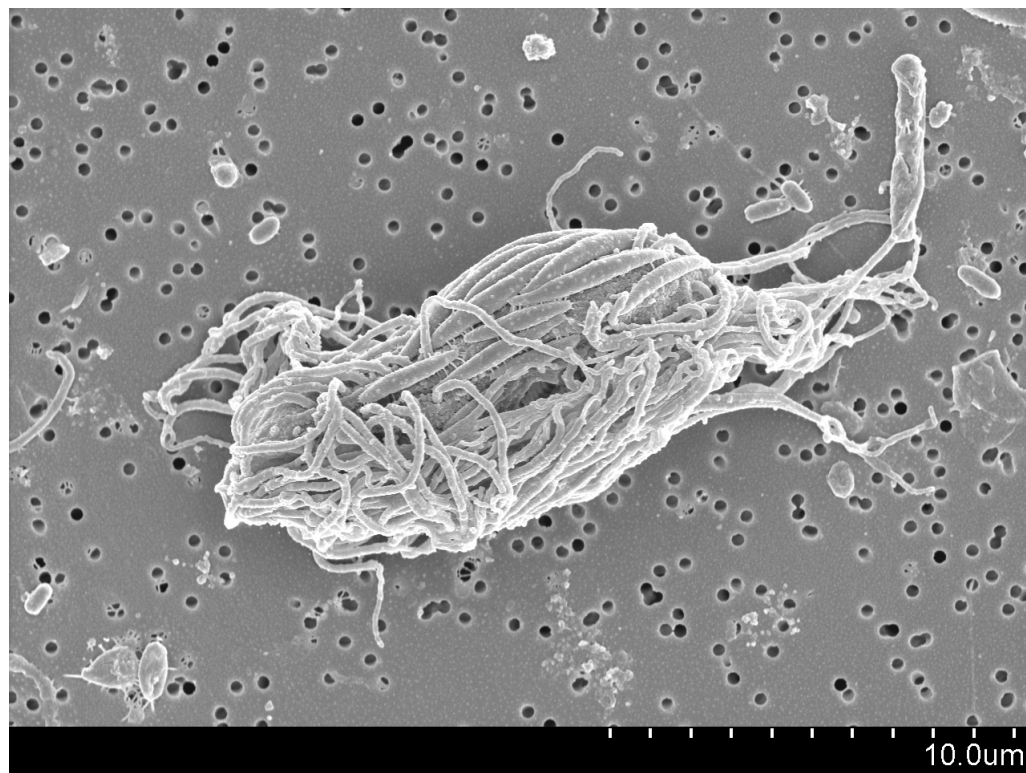

Supplement: Additional file 1 — Scanning electron micrograph of an unidentified ciliate isolated from anoxic, sulfidic waters of the Cariaco Basin. Figure S1. The ciliate in the picture, isolated from anoxic waters of the Cariaco basin, is covered with bacterial ectosymbionts. Protists with bacterial ectosymbionts are frequently recovered from sulfidic waters of both, the Cariaco Basin as well as the Framvaren Fjord. It is not unlikely that these as yet unidentified bacteria may play a role as an adaptive mechanisms for some protists to thrive in anoxic sulfidic environments. This picture is courtesy of Orsi W., Edgcomb V., Hohemann T. and Epstein S.S. as part of a study on bacterial ectosymbionts on protists from the Cariaco Basin (Orsi et al., in preparation for publication). [file 1741-7007-7-72-S1.pdf]

Supplemental Figure S2

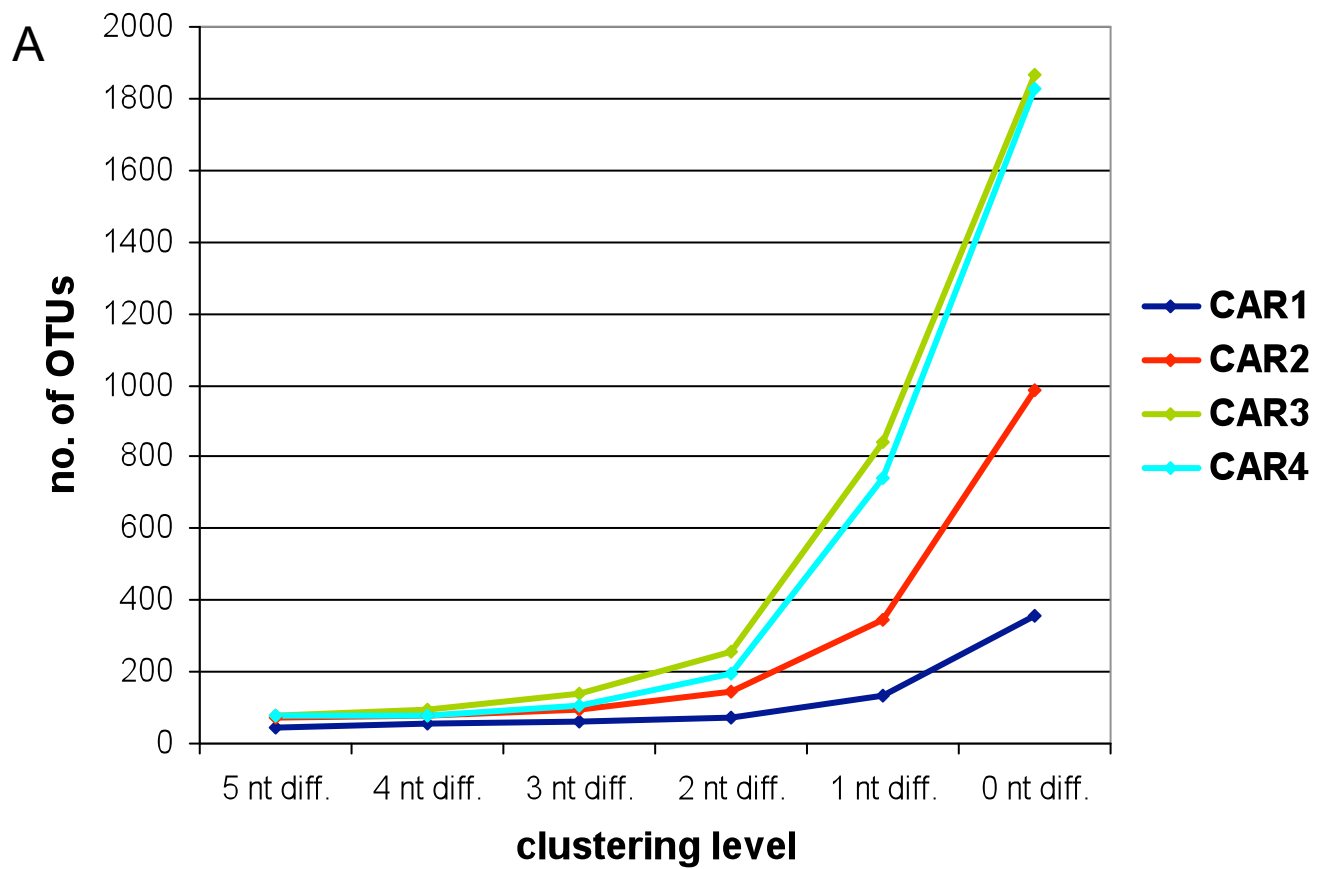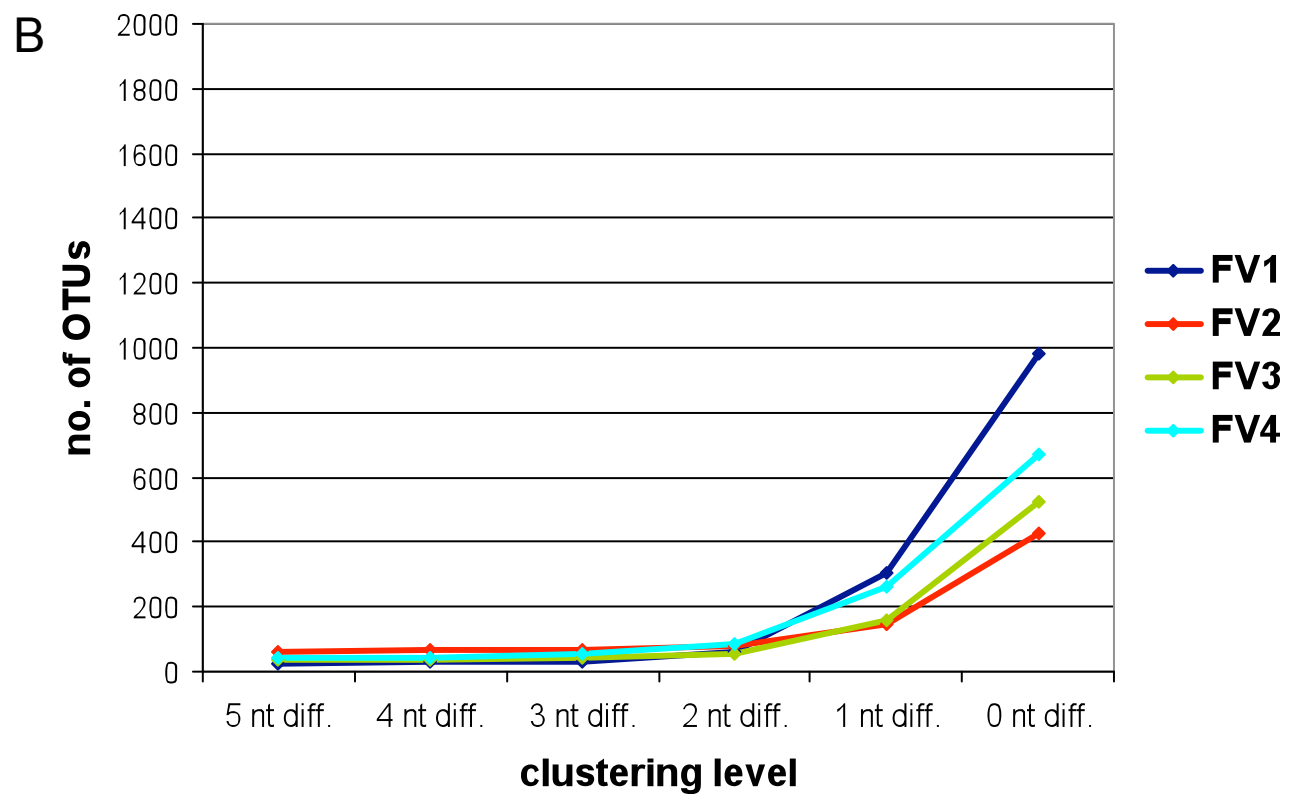

Supplement: Additional file 3 — Numbers of unique metazoan operational taxonomic units. Figure S2. Number of unique metazoan operational taxonomic units (OTUs) obtained from four samples of the Caribbean Cariaco Basin (CAR1-4, Figure S2-A) and four samples of the Norwegian Framvaren Fjord (FV1-4, Figure S2-B) at different levels of nucleotide differences. Tags were clustered at nt differences zero to five differences as described in pipeline 2 of the sequence data processing paragraph in the methods section. A difference can be an insertion or a mutation necessary to align the two sequences. At k differences, two tags having k or fewer differences are placed in the same cluster; if they have more than k differences, they are in two different clusters. [file 1741-7007-7-72-S3.pdf]
