# Supplementary material for: Massively parallel tag sequencing reveals the complexity of anaerobic marine protistan communities
Source: BMC Biol. 2009 Nov 3;7:72. doi: 10.1186/1741-7007-7-72 (PMC2777867; doi:10.1186/1741-7007-7-72)
Supplement: Additional file 2 — Taxonomy and proportion of abundant metazoan operational taxonomic units. Table S1. Taxonomy and proportion of abundant metazoan operational taxonomic units (OTUs) accounting for at least 1% of all metazoan OTUs of a specific amplicon library from four anoxic water samples from the Caribbean Cariaco deep-sea basin (CAR1-4) and four anoxic water samples of the Norwegian Framvaren Fjord (FV1-4). OTUs were established based on identical best GenBank hit. For each OTU the best GenBank match is given (accession no., organism description, and taxonomy), as well as the number of total and unique tags. Unique tags are tags clustered at 0 differences. [file 1741-7007-7-72-S2.doc]

**Table S1.** Taxonomy and proportion of abundant metazoan OTUs accounting for at least 1% of all metazoan OTUs of a specific amplicon library from four anoxic water samples from the Caribbean Cariaco deep-sea basin (CAR1-4) and four anoxic water samples of the Norwegian Framvaren Fjord (FV1-4). OTUs were established based on identical best GenBank hit. For each OTU the best GenBank match is given (accession no., organism description, and taxonomy), as well as the number of total and unique tags. Unique tags are tags clustered at 0 differences.

| **sample** | **OTU no.** | **best GenBank hit** | | | **no. total tags** | **no. unique tags** |
| --- | --- | --- | --- | --- | --- | --- |
|  |  | **acc. no.** | **organism** | **taxonomy** |  |  |
| **FV1** | 1 | U50971 | *Polydora ciliata* | Polychaeta | 734 | 91 |
|  | 2 | AY665127 | uncultured eukaryote | Copepoda | 8652 | 400 |
|  | 3 | DQ369014 | uncultured marine eukaryote | Copepoda | 1465 | 156 |
|  | 4 | AY665125 | uncultured eukaryote | Copepoda | 2443 | 214 |
|  | 5 | DQ297715 | *Ploesoma truncatus* | Rotifera | 603 | 68 |
|  |  |  |  |  |  |  |
| **FV2** | 1 | U50971 | *Polydora ciliata* | Polychaeta | 1407 | 181 |
|  | 2 | AY665127 | uncultured eukaryote | Copepoda | 304 | 53 |
|  | 3 | L81939 | *Calanus pacificus* | Copepoda | 86 | 19 |
|  | 4 | AY665125 | uncultured eukaryote | Copepoda | 401 | 54 |
|  | 5 | FJ009098 | *Steginoporella truncata* | Bryozoa | 32 | 5 |
|  | 6 | AF102898 | *Anaperus tvaerminnensis* | Platyhelminthes | 44 | 8 |
|  | 7 | DQ297715 | *Ploesoma truncatus* | Rotifera | 26 | 4 |
|  |  |  |  |  |  |  |
| **FV3** | 1 | U50971 | *Polydora ciliata* | Polychaeta | 835 | 116 |
|  | 2 | AY665127 | uncultured eukaryote | Copepoda | 767 | 97 |
|  | 3 | L81939 | *Calanus pacificus* | Copepoda | 298 | 74 |
|  | 4 | AY665125 | uncultured eukaryote | Copepoda | 1084 | 139 |
|  | 5 | FJ009098 | *Steginoporella truncata* | Bryozoa | 46 | 13 |
|  | 6 | AY962535 | *Junceella aquamata* | Cnidaria | 83 | 26 |
|  | 7 | L33455 | *Mytilus trossulus* | Mollusca | 36 | 3 |
|  |  |  |  |  |  |  |
| **FV4** | 1 | U50971 | *Polydora ciliata* | Polychaeta | 379 | 90 |
|  | 2 | AY665127 | uncultured eukaryote | Copepoda | 155 | 41 |
|  | 3 | L81939 | *Calanus pacificus* | Copepoda | 372 | 77 |
|  | 4 | AY665125 | uncultured eukaryote | Copepoda | 129 | 27 |
|  | 5 | AY216698 | *Lysiphlebus testaceipes* | Insecta | 4857 | 328 |

| **sample** | **OTU no.** | **best GenBank hit** | | | **no. total tags** | **no. unique tags** |
| --- | --- | --- | --- | --- | --- | --- |
|  |  | **acc. no.** | **organism** | **taxonomy** |  |  |
| **CAR1** | 1 | L81939 | *Calanus pacificus* | Copepoda | 94 | 18 |
|  | 2 | DQ369014 | uncultured marine eukaryote | Copepoda | 48 | 14 |
|  | 3 | AY937360 | *Lensia conoidea* | Cnidaria | 78 | 13 |
|  | 4 | DQ080014 | *Lilyopsis rosea* | Cnidaria | 28 | 11 |
|  | 5 | AY937340 | *Agalma elegans* | Cnidaria | 63 | 9 |
|  | 6 | DQ080012 | *Physonect* sp. E YPM-35826 | Cnidaria | 127 | 23 |
|  | 7 | AY920756 | *Liriope tetraphylla* | Cnidaria | 71 | 14 |
|  | 8 | AY665135 | uncultured eukaryote | Cnidaria | 1941 | 181 |
|  |  |  |  |  |  |  |
| **CAR2** | 1 | U50971 | *Polydora ciliata* | Polychaeta | 245 | 54 |
|  | 2 | AY665127 | uncultured eukaryote | Copepoda | 465 | 81 |
|  | 3 | L81939 | *Calanus pacificus* | Copepoda | 1209 | 188 |
|  | 4 | DQ369014 | uncultured marine eukaryote | Copepoda | 95 | 21 |
|  | 5 | AY665125 | uncultured eukaryote | Copepoda | 260 | 62 |
|  | 6 | FJ009098 | *Steginoporella truncata* | Bryozoa | 108 | 20 |
|  | 7 | AY962535 | *Junceella aquamata* | Cnidaria | 120 | 23 |
|  | 8 | EU305493 | *Eutima sapinhoa* | Cnidaria | 144 | 25 |
|  | 9 | AY937360 | *Lensia conoidea* | Cnidaria | 1421 | 171 |
|  | 10 | AY920756 | *Liriope tetraphylla* | Cnidaria | 679 | 100 |
|  | 11 | AY665135 | uncultured eukaryote | Cnidaria | 409 | 76 |
|  | 12 | AY788914 | uncultured marine eukaryote | Ctenophora | 71 | 6 |
|  |  |  |  |  |  |  |
| **CAR3** | 1 | AM490294 | *Sida crystallina* | Branchiopoda | 4011 | 355 |
|  | 2 | L34043 | *Bythotrephes cederstroemi* | Branchiopoda | 4253 | 629 |
|  | 3 | L81939 | *Calanus pacificus* | Copepoda | 894 | 179 |
|  | 4 | AY962535 | *Junceella aquamata* | Cnidaria | 4699 | 346 |
|  | 5 | AY920756 | *Liriope tetraphylla* | Cnidaria | 956 | 131 |
|  |  |  |  |  |  |  |
| **CAR4** | 1 | L81939 | *Calanus pacificus* | Copepoda | 2279 | 267 |
|  | 2 | AY937360 | *Lensia conoidea* | Cnidaria | 2506 | 255 |
|  | 3 | DQ080014 | *Lilyopsis rosea* | Cnidaria | 8378 | 585 |
|  | 4 | AY937351 | *Rhizophysa eysenhardti* | Cnidaria | 450 | 93 |
|  | 5 | AY937324 | *Nanomia bijuga* | Cnidaria | 1826 | 235 |
|  | 6 | AY920756 | *Liriope tetraphylla* | Cnidaria | 784 | 133 |
