# Supplementary material for: Massively parallel tag sequencing reveals the complexity of anaerobic marine protistan communities
Source: BMC Biol. 2009 Nov 3;7:72. doi: 10.1186/1741-7007-7-72 (PMC2777867; doi:10.1186/1741-7007-7-72)
Supplement: Additional file 4 — Relative contribution of metazoan operational taxonomic units to total eukaryote operational taxonomic units. Table S2. Relative contribution of metazoan operational taxonomic units (OTUs) to total eukaryote OTUs when clustered at 5 nt differences (OTUs5 nt) in the Franvaren Fjord (FV1-FV4) and the Cariaco Basin (CAR1-CAR4) amplicon libraries. [file 1741-7007-7-72-S4.doc]

**Table S2.** Relative contribution of metazoan OTUs to total eukaryote OTUs when clustered at 5 nt differences (OTUs_5nt_) in the Framvaren Fjord (FV1-FV4) and the Cariaco Basin (CAR1-CAR4) amplicon libraries.

|  | FV1 | FV2 | FV3 | FV4 | CAR1 | CAR2 | CAR3 | CAR4 |
| --- | --- | --- | --- | --- | --- | --- | --- | --- |
| relative contribution of metazoan OTUs_5nt_ to total eukaryote OTUs_5nt_ (%) | 7.00 | 6.27 | 5.30 | 6.18 | 3.96 | 6.55 | 11.45 | 11.11 |
