# Supplementary material for: Massively parallel tag sequencing reveals the complexity of anaerobic marine protistan communities
Source: BMC Biol. 2009 Nov 3;7:72. doi: 10.1186/1741-7007-7-72 (PMC2777867; doi:10.1186/1741-7007-7-72)
Supplement: Additional file 5 — Relative taxonomic distribution of unique protistan and fungal V9 tags. Table S3. Accompanying data to Figure 4. Relative taxonomic distribution of unique protistan and fungal V9 tags generated from four anoxic water samples of the Caribbean Cariaco deep-sea basin (CAR1-4) and from four anoxic water samples of the Norwegian Framvaren Fjord (FV1-4). Phylum-based assignment; phyla that were represented by a proportion ≥1% of all unique tags in at least one of the eight libraries used for 454 sequencing is shown. The category "others" denotes tags that could not be assigned to a taxonomic entity based on an 80% BLASTn similarity threshold and tags which fell into other phyla or taxon groups but were represented by <1% of the unique tags in all of the eight PCR amplicon libraries used for 454 sequencing. Table S4. Accompanying data to Figure 5. Relative taxonomic distribution of unique protistan and fungal V9 tags generated from four anoxic water samples of the Caribbean Cariaco deep-sea basin (CAR1-4) and from four anoxic water samples of the Norwegian Framvaren Fjord (FV1-4) within the Dinozoa. Table S5. Accompanying data to Figure 6. Relative taxonomic distribution of unique protistan and fungal V9 tags generated from four anoxic water samples of the Caribbean Cariaco deep-sea basin (CAR1-4) and from four anoxic water samples of the Norwegian Framvaren Fjord (FV1-4) within the Ciliophora. Table S6. Accompanying data to Figure 7. Relative taxonomic distribution of unique protistan and fungal V9 tags generated from four anoxic water samples of the Caribbean Cariaco deep-sea basin (CAR1-4) and from four anoxic water samples of the Norwegian Framvaren Fjord (FV1-4) within the Bacillariophyta. Table S7. Accompanying data to Figure 8. Relative taxonomic distribution of unique protistan and fungal V9 tags generated from four anoxic water samples of the Caribbean Cariaco deep-sea basin (CAR1-4) and from four anoxic water samples of the Norwegian Framvaren Fjord (FV1-4) withi [file 1741-7007-7-72-S5.doc]

**Table S3:** Accompanying data to Figure 4. Relative taxonomic distribution of unique protistan and fungal V9 tags generated from four anoxic water samples of the Caribbean Cariaco deep-sea basin (CAR1-4) and from four anoxic water samples of the Norwegian Framvaren Fjord (FV1-4). Phylum-based assignment; phyla that were represented by a proportion ≥1% of all unique tags in at least one of the eight libraries used for 454 sequencing is shown. The category “others” denotes tags that could not be assigned to a taxonomic entity based on an 80% BLASTn similarity threshold and tags which fell into other phyla or taxon groups but were represented by <1% of the unique tags in all of the eight PCR amplicon libraries used for 454 sequencing.

|  | **FV1** | **FV2** | **FV3** | **FV4** | **CAR1** | **CAR2** | **CAR3** | **CAR4** |
| --- | --- | --- | --- | --- | --- | --- | --- | --- |
| unassignable | 4.38 | 6.49 | 9.13 | 3.51 | 6.82 | 9.84 | 21.07 | 13.16 |
| Ciliophora | 22.06 | 29.42 | 11.75 | 15.58 | 2.63 | 11.28 | 4.96 | 14.21 |
| Dinozoa | 42.39 | 14.66 | 30.53 | 14.82 | 30.79 | 25.78 | 33.75 | 23.85 |
| Apicomplexa | 0.24 | 0.43 | 1.17 | 1.77 | 0.41 | 1.02 | 0.28 | 0.78 |
| Ascomycota | 0.30 | 0.78 | 0.43 | 0.38 | 0.12 | 1.19 | 0.69 | 0.55 |
| Basidiomycota | 0.00 | 0.66 | 0.65 | 1.39 | 0.16 | 1.00 | 1.87 | 1.19 |
| Choanozoa | 0.60 | 0.47 | 1.30 | 3.72 | 0.12 | 0.30 | 0.28 | 0.73 |
| Cryptophyta | 7.32 | 4.28 | 3.98 | 2.24 | 0.23 | 1.89 | 0.65 | 1.05 |
| Kathablepharidae (incertae sedis) | 1.37 | 0.45 | 0.25 | 0.27 | 0.05 | 0.15 | 0.08 | 0.09 |
| Cercozoa | 1.64 | 7.00 | 2.74 | 5.42 | 0.14 | 2.57 | 0.89 | 1.83 |
| Radiolaria (Polycystinea) | 0.00 | 0.00 | 0.00 | 0.00 | 31.50 | 6.04 | 3.50 | 7.13 |
| Granuloreticulosa | 0.00 | 0.14 | 0.00 | 0.09 | 0.00 | 0.02 | 0.12 | 2.33 |
| Bacillariophyta | 3.07 | 4.28 | 6.72 | 8.59 | 1.88 | 11.66 | 7.69 | 5.76 |
| Heterokontophyta | 1.70 | 8.59 | 4.93 | 11.03 | 0.32 | 2.14 | 0.53 | 2.88 |
| Labyrinthulomycota | 0.21 | 0.82 | 0.71 | 0.58 | 0.48 | 1.46 | 0.57 | 0.46 |
| other stramenopiles | 2.35 | 1.98 | 2.13 | 1.19 | 1.00 | 1.38 | 1.14 | 1.10 |
| Haptophyta | 0.48 | 2.24 | 0.83 | 1.88 | 0.41 | 0.87 | 0.77 | 1.60 |
| Chlorophyta | 0.86 | 3.71 | 5.12 | 2.53 | 0.28 | 2.06 | 1.46 | 2.56 |
| environmental samples | 9.85 | 8.87 | 14.46 | 22.49 | 21.00 | 15.75 | 17.57 | 16.31 |
| others | 1.19 | 4.71 | 3.18 | 2.53 | 1.63 | 3.63 | 2.11 | 2.47 |

**Table S4:** Accompanying data to Figure 5. Relative taxonomic distribution of unique protistan and fungal V9 tags generated from four anoxic water samples of the Caribbean Cariaco deep-sea basin (CAR1-4) and from four anoxic water samples of the Norwegian Framvaren Fjord (FV1-4) within the Dinozoa.

|  | **FV1** | **FV2** | **FV3** | **FV4** | **CAR1** | **CAR2** | **CAR3** | **CAR4** |
| --- | --- | --- | --- | --- | --- | --- | --- | --- |
| Blastodiniales | 0.00 | 0.00 | 0.00 | 0.00 | 0.46 | 0.91 | 5.66 | 0.19 |
| Dinophysiales | 1.05 | 0.85 | 0.91 | 0.76 | 0.29 | 0.41 | 2.05 | 0.96 |
| Gonyaulacales | 7.44 | 11.83 | 20.12 | 5.45 | 1.21 | 9.97 | 20.36 | 4.99 |
| Gymnodiniales | 22.40 | 26.20 | 29.67 | 23.94 | 19.63 | 25.70 | 14.10 | 19.58 |
| Noctilucales | 0.00 | 0.00 | 0.00 | 0.00 | 0.12 | 0.99 | 0.36 | 0.38 |
| Oxyrrhinales | 0.63 | 18.17 | 1.52 | 6.36 | 0.00 | 1.65 | 0.36 | 3.84 |
| Peridiniales | 19.17 | 14.93 | 21.95 | 34.55 | 11.72 | 27.18 | 27.59 | 24.57 |
| Phytodiniales | 0.00 | 0.00 | 0.00 | 0.00 | 0.12 | 0.00 | 0.00 | 0.00 |
| Prorocentrales | 21.70 | 8.45 | 10.77 | 8.18 | 13.63 | 10.87 | 11.33 | 14.01 |
| Pyrocystales | 0.00 | 0.00 | 0.30 | 0.15 | 0.00 | 0.25 | 0.00 | 0.38 |
| Suessiales | 0.21 | 1.55 | 0.81 | 4.55 | 4.73 | 1.48 | 0.96 | 2.50 |
| Syndiniales | 2.25 | 3.94 | 6.10 | 5.00 | 13.63 | 7.41 | 4.94 | 10.56 |
| Thoracosphaerales | 0.00 | 0.28 | 0.81 | 0.76 | 0.69 | 0.74 | 0.60 | 0.19 |
| environmental samples | 3.37 | 2.39 | 1.12 | 2.58 | 4.10 | 2.64 | 1.57 | 1.73 |
| unclassified Dinozoa | 21.77 | 11.41 | 5.89 | 7.73 | 29.68 | 9.80 | 10.12 | 16.12 |

**Table S5:** Accompanying data to Figure 6. Relative taxonomic distribution of unique protistan and fungal V9 tags generated from four anoxic water samples of the Caribbean Cariaco deep-sea basin (CAR1-4) and from four anoxic water samples of the Norwegian Framvaren Fjord (FV1-4) within the Ciliophora.

|  | **FV1** | **FV2** | **FV3** | **FV4** | **CAR1** | **CAR2** | **CAR3** | **CAR4** |
| --- | --- | --- | --- | --- | --- | --- | --- | --- |
| Colpodea | 0.40 | 0.00 | 0.52 | 0.14 | 22.30 | 1.88 | 0.00 | 0.64 |
| Litostomatea | 13.36 | 13.23 | 16.01 | 6.32 | 2.70 | 15.23 | 16.39 | 14.47 |
| Nassophorea | 0.00 | 0.49 | 1.05 | 0.14 | 1.35 | 0.19 | 0.00 | 0.96 |
| Oligohymenophorea | 6.61 | 37.72 | 29.13 | 38.22 | 9.46 | 31.77 | 18.03 | 33.76 |
| Phyllopharyngea | 0.00 | 0.91 | 0.00 | 0.72 | 0.00 | 0.19 | 0.00 | 0.64 |
| Prostomatea | 5.94 | 2.80 | 4.20 | 2.87 | 0.68 | 2.44 | 3.28 | 4.18 |
| Spirotrichea | 56.28 | 28.83 | 25.20 | 33.48 | 56.08 | 33.27 | 50.00 | 32.80 |
| Heterotrichea | 2.97 | 1.12 | 19.16 | 1.87 | 0.68 | 5.45 | 2.46 | 2.57 |
| environmental samples | 14.44 | 14.91 | 4.72 | 16.24 | 6.76 | 9.59 | 9.84 | 9.97 |

**Table S6:** Accompanying data to Figure 7. Relative taxonomic distribution of unique protistan and fungal V9 tags generated from four anoxic water samples of the Caribbean Cariaco deep-sea basin (CAR1-4) and from four anoxic water samples of the Norwegian Framvaren Fjord (FV1-4) within the Bacillariophyta.

|  | **FV1** | **FV2** | **FV3** | **FV4** | **CAR1** | **CAR2** | **CAR3** | **CAR4** |
| --- | --- | --- | --- | --- | --- | --- | --- | --- |
| Bacillariophycidae | 3.92 | 25.48 | 10.09 | 9.38 | 37.74 | 35.09 | 30.16 | 13.49 |
| Eunotiophycidae | 0.98 | 2.40 | 0.46 | 0.52 | 0.94 | 0.36 | 0.00 | 0.79 |
| Biddulphiophycidae | 0.00 | 0.96 | 0.00 | 0.00 | 2.83 | 2.55 | 2.12 | 0.79 |
| Chaetocerotophycidae | 46.08 | 31.73 | 39.91 | 42.71 | 13.21 | 20.36 | 19.58 | 28.57 |
| Corethrophycidae | 0.00 | 2.40 | 4.59 | 0.26 | 1.89 | 2.00 | 1.59 | 1.59 |
| Coscinodiscophycidae | 0.00 | 0.00 | 0.46 | 0.26 | 0.00 | 0.18 | 3.70 | 0.79 |
| Cymatosirophycidae | 0.00 | 0.00 | 0.46 | 0.26 | 0.94 | 0.55 | 2.65 | 0.79 |
| Lithodesmiophycidae | 0.00 | 0.00 | 0.00 | 0.00 | 0.94 | 0.55 | 0.00 | 0.00 |
| Rhizosoleniophycidae | 0.00 | 0.48 | 0.92 | 0.52 | 6.60 | 8.55 | 4.23 | 5.56 |
| Thalassiosirophycidae | 6.86 | 13.46 | 22.02 | 13.02 | 25.47 | 20.91 | 29.10 | 35.71 |
| Fragilariophycidae | 0.98 | 0.96 | 0.92 | 1.82 | 4.72 | 1.82 | 3.17 | 3.17 |
| environmental samples | 41.18 | 22.12 | 20.18 | 31.25 | 4.72 | 7.09 | 3.70 | 8.73 |

**Table S7:** Accompanying data to Figure 8. Relative taxonomic distribution of unique protistan and fungal V9 tags generated from four anoxic water samples of the Caribbean Cariaco deep-sea basin (CAR1-4) and from four anoxic water samples of the Norwegian Framvaren Fjord (FV1-4) within the Chlorophyta.

|  | **FV1** | **FV2** | **FV3** | **FV4** | **CAR1** | **CAR2** | **CAR3** | **CAR4** |
| --- | --- | --- | --- | --- | --- | --- | --- | --- |
| Chlamydomonadales | 3.45 | 6.11 | 9.04 | 3.54 | 0.00 | 2.15 | 22.86 | 3.70 |
| Chlorococcales | 0.00 | 0.00 | 0.00 | 5.31 | 0.00 | 0.00 | 0.00 | 0.00 |
| Sphaeropleales | 0.00 | 1.67 | 0.60 | 0.88 | 12.50 | 3.23 | 0.00 | 1.85 |
| Chlorodendrales | 0.00 | 28.89 | 0.00 | 13.27 | 0.00 | 15.05 | 0.00 | 12.96 |
| Mamiellales | 17.24 | 2.78 | 1.81 | 9.73 | 25.00 | 7.53 | 8.57 | 22.22 |
| Pseudoscourfieldiales | 3.45 | 1.67 | 2.41 | 0.88 | 12.50 | 8.60 | 20.00 | 11.11 |
| Pyramimonadales | 55.17 | 7.22 | 9.64 | 17.70 | 18.75 | 11.83 | 8.57 | 16.67 |
| environmental Prasinophyceae | 0.00 | 4.44 | 1.81 | 0.88 | 6.25 | 8.60 | 5.71 | 3.70 |
| Chlorellales | 0.00 | 0.56 | 0.00 | 0.88 | 12.50 | 5.38 | 14.29 | 3.70 |
| Choricystis | 10.34 | 34.44 | 69.88 | 38.94 | 0.00 | 31.18 | 11.43 | 22.22 |
| Microthamniales | 0.00 | 0.56 | 0.60 | 1.77 | 0.00 | 0.00 | 0.00 | 0.00 |
| Picochlorum | 0.00 | 1.67 | 0.60 | 0.00 | 0.00 | 0.00 | 0.00 | 0.00 |
| Acrosiphoniales | 0.00 | 0.56 | 0.00 | 0.00 | 0.00 | 0.00 | 0.00 | 0.00 |
| Bryopsidales | 0.00 | 0.56 | 0.00 | 0.00 | 0.00 | 0.00 | 0.00 | 0.00 |
| environmental samples | 10.34 | 8.89 | 3.61 | 6.19 | 12.50 | 6.45 | 8.57 | 1.85 |

**Table S8:** Accompanying data to Figure 9. Relative taxonomic distribution of unique protistan and fungal V9 tags generated from four anoxic water samples of the Caribbean Cariaco deep-sea basin (CAR1-4) and from four anoxic water samples of the Norwegian Framvaren Fjord (FV1-4) within the Heterokontophyta.

|  | **FV1** | **FV2** | **FV3** | **FV4** | **CAR1** | **CAR2** | **CAR3** | **CAR4** |
| --- | --- | --- | --- | --- | --- | --- | --- | --- |
| Bicosoecida | 3.41 | 7.01 | 13.36 | 4.28 | 6.90 | 12.06 | 11.43 | 5.88 |
| Bolidophyceae | 3.41 | 1.65 | 2.76 | 0.93 | 20.69 | 2.13 | 0.00 | 1.18 |
| Chrysophyceae | 67.05 | 67.42 | 53.46 | 84.94 | 37.93 | 54.61 | 60.00 | 61.18 |
| Dictyochophyceae | 25.00 | 18.56 | 20.28 | 6.69 | 17.24 | 17.02 | 5.71 | 12.94 |
| Eustigmatophyceae | 1.14 | 0.62 | 3.69 | 0.19 | 0.00 | 0.71 | 2.86 | 1.18 |
| Pelagophyceae | 0.00 | 3.51 | 1.84 | 1.30 | 10.34 | 2.84 | 14.29 | 3.53 |
| Phaeophyceae | 0.00 | 0.21 | 1.84 | 0.00 | 3.45 | 2.84 | 2.86 | 11.76 |
| Raphidophyceae | 0.00 | 0.00 | 0.92 | 0.37 | 3.45 | 7.09 | 2.86 | 0.00 |
| Xanthophyceae | 0.00 | 1.03 | 1.84 | 1.30 | 0.00 | 0.71 | 0.00 | 2.35 |
